# Granulomatous variant of scleromyxedema successfully treated with topical ruxolitinib, dapsone and intravenous immunoglobulin

**DOI:** 10.1016/j.jdcr.2023.10.010

**Published:** 2023-11-01

**Authors:** Donglin Zhang, Kimberly Sable, Allison Miller, Molly Hinshaw, Timothy Schmidt, Bridget E. Shields

**Affiliations:** a Department of Dermatology, University of Wisconsin School of Medicine and Public Health, Madison, Wisconsin; b Department of Hematology and Oncology, University of Wisconsin School of Medicine and Public Health, Madison, Wisconsin

**Keywords:** cutaneous mucinosis, granulomatous scleromyxedema, JAK inhibitor, sclerodermoid lichen myxedematosus, scleromyxedema

## Background

Scleromyxedema (SM) is a progressive, generalized cutaneous mucinosis of unknown etiology. SM is a rare entity with limited reports describing the spectrum of clinical and histologic findings. Clinically, SM is characterized by a generalized papular and sclerodermoid eruption and is commonly associated with an IgG-λ paraproteinemia.^1^ Histologic examination classically reveals (1) mucin deposition with in the upper and midreticular dermis, (2) fibroblast proliferation, and (3) fibrosis.^2^ Other mucinoses such as cutaneous myxedema related to thyroid dysfunction must be excluded. First-line therapy is generally with intravenous immunoglobulin (IVIG) given its favorable efficacy and tolerability profile among patients with SM, with thalidomide and/or systemic corticosteroids as second-line treatment options.^3^ We present an unusual case of granulomatous scleromyxedema with symptomatic improvement following treatment with a topical Janus kinase inhibitor (JAKi) and IVIG.

## Case report

**Fig 1:**
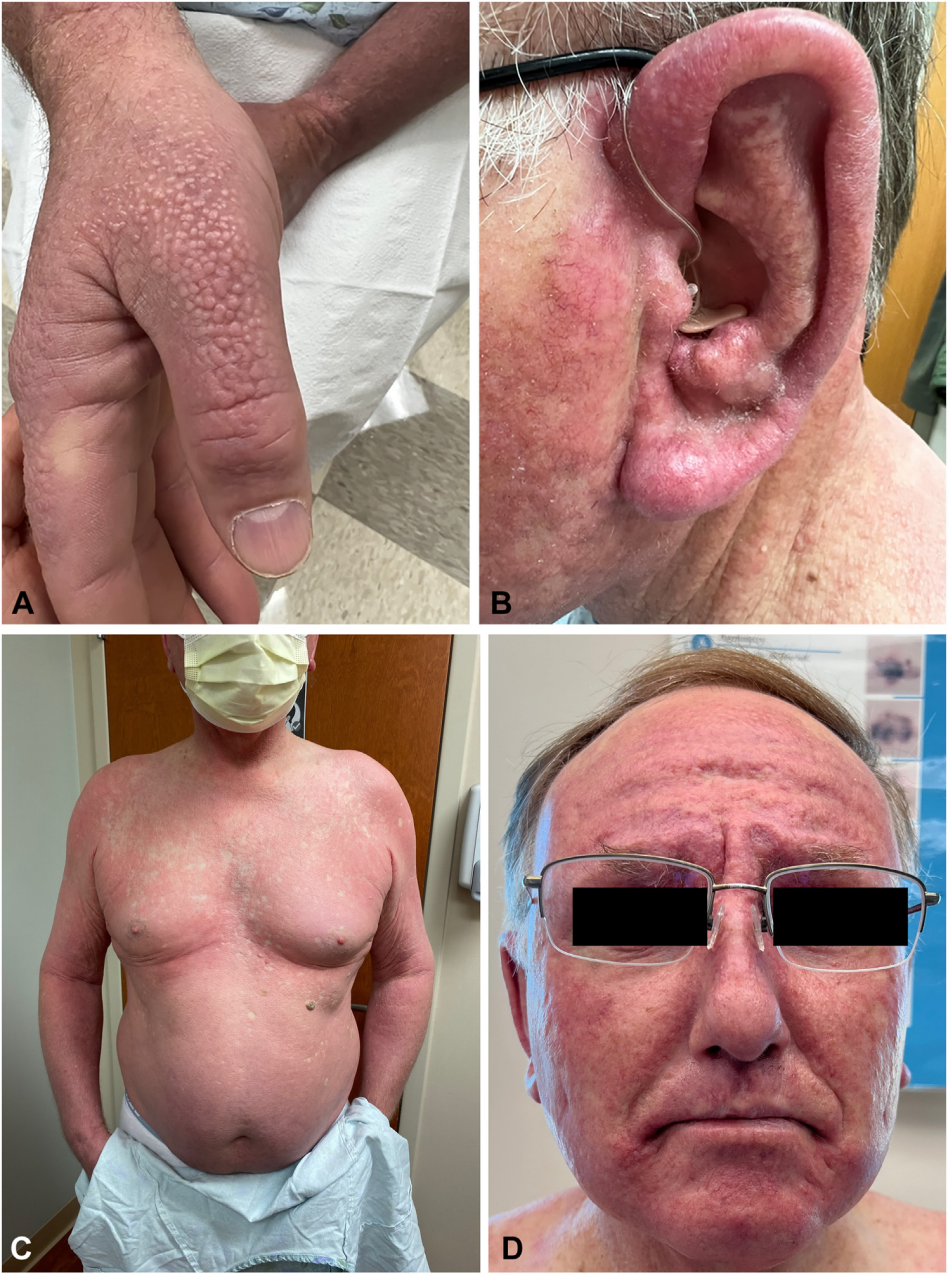
**A**, Small, pink and flesh-colored, flat-topped papules densely grouped on the dorsal aspect of the hand. **B**, Erythematous, indurated plaques covering the ear, with mucinous papules on the antihelix and antitragus. **C**, Diffuse, indurated red-brown papules coalescing into plaques involving his entire body. **D**, Prominent erythema and induration of the forehead, glabella, and perioral face with evolving leonine facies.

A man in his 60s with a past medical history of subclinical hypothyroidism and hyperlipidemia developed papules coalescing into diffuse plaques on the extremities, trunk, ears, and face over the course of 4 months. Multiple biopsies of affected skin exhibited interstitial inflammation with increased dermal mucin in areas of granulomatous dermatitis, resulting in a diagnosis of generalized granuloma annulare (GA). Failure to improve on topical triamcinolone, oral prednisone, and narrow-band UV-B therapy over 2 years led to the initiation of oral hydroxychloroquine and referral to our institution. At the time of presentation to our tertiary care center, physical examination revealed diffuse, red-brown papules coalescing into plaques involving his entire body with islands of sparing on his back and bilateral lower portion of the legs along with mucinous papules on bilateral ears and dorsal aspect of the hands. The forehead and perioral face were found to have significant induration with evolving leonine facies (Fig 1). The skin lesions were associated with persistent pruritus interfering with his quality of life, and tenderness isolated to the ears that worsened with pressure. Review of systems was otherwise negative.

**Fig 2:**
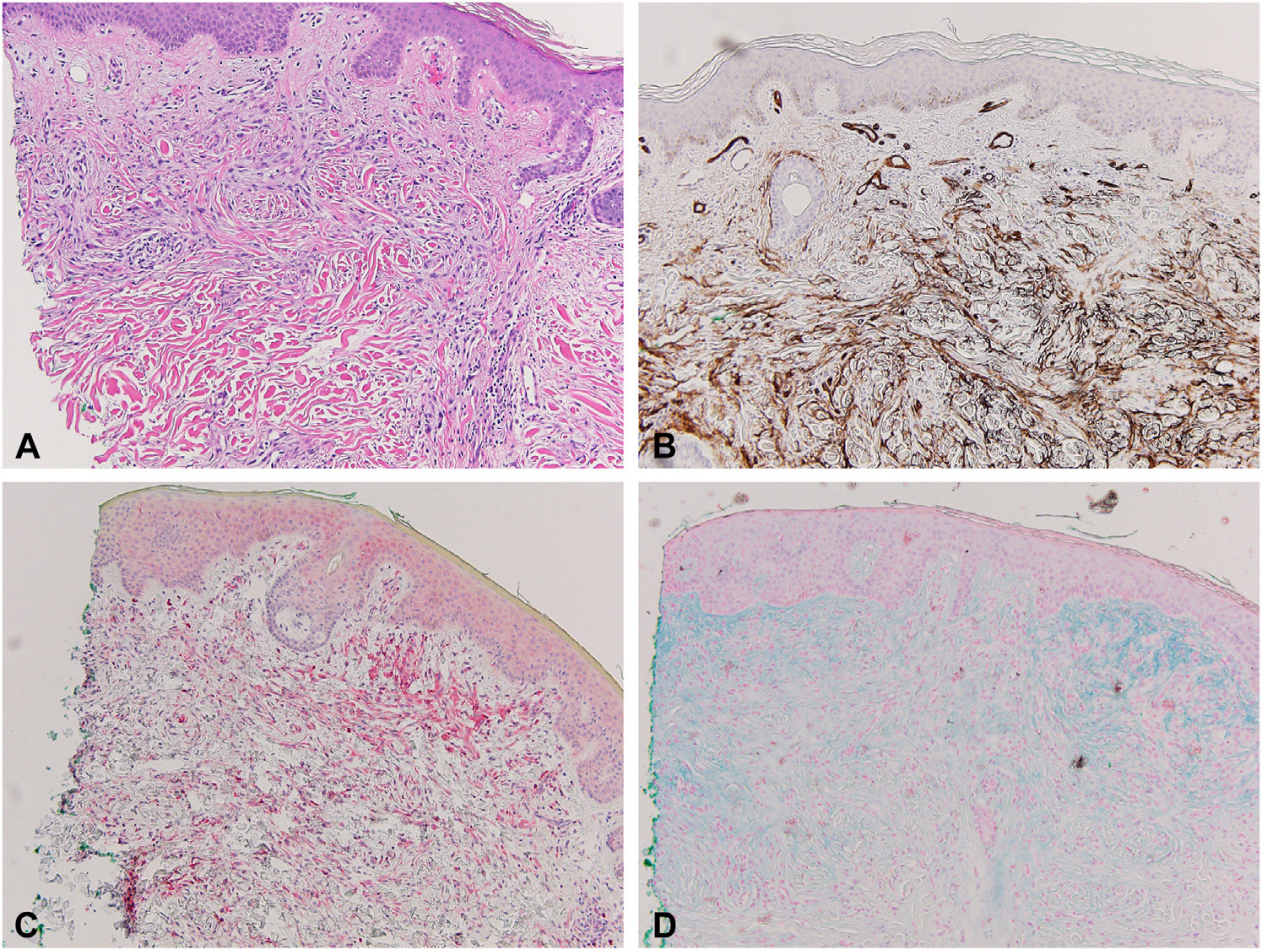
Punch biopsy of skin of left shoulder (×100). **A**, Hematoxylin-eosin revealing an interstitial and vaguely palisaded proliferation of variably spindled and epithelioid-shaped cells. **B**, CD34 stain revealed spindled fibroblasts. **C**, CD68 stains revealed epithelioid histiocytes. **D**, Alcian blue stain revealed increased dermal mucin.

**Fig 3:**
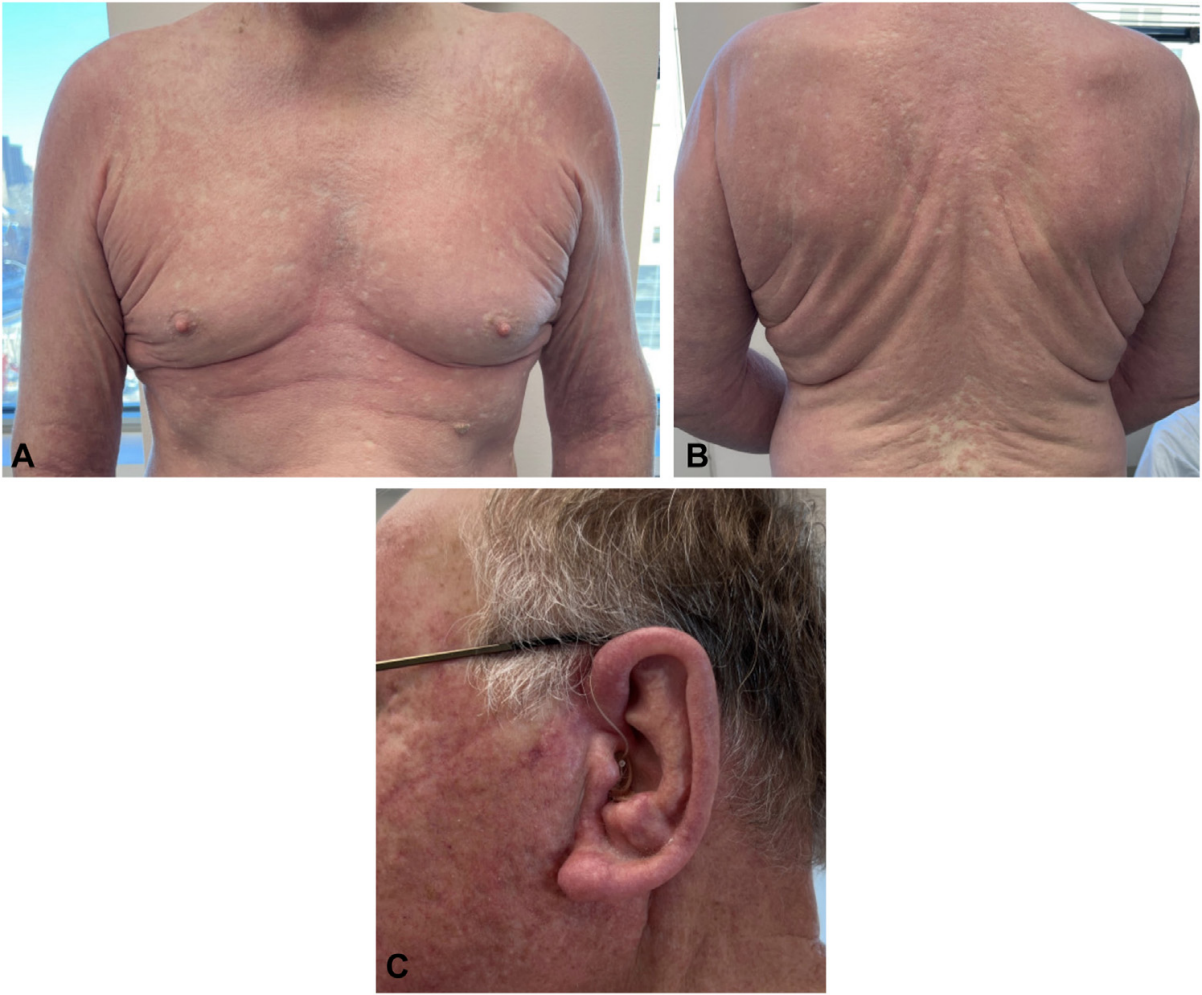
Clinical improvement following topical ruxolitinib. Reduction in skin redness and induration of the **(A)** central chest, **(B)** upper portion of the back, and **(C)** left ear.

Laboratory evaluation revealed an M-spike (0.4 g/dL) on serum protein electrophoresis, and serum protein immunofixation confirmed an IgG-λ light chain monoclonal gammopathy. Urine protein electrophoresis and immunofixation were within normal limits. Serum free kappa and lambda light chains and kappa-lambda ratio were normal. A slightly elevated thyroid stimulating hormone (5.87 mIU/mL) with a normal free T4 (1.0 ng/dL) was identified. The patient was up to date on age-appropriate cancer screenings. Histologic examination of 4-mm punch biopsies of the left shoulder and left side of the mid-back revealed variably interstitial and vaguely palisaded histiocytic infiltrate in the papillary dermis with increased dermal mucin, with immunohistochemical demonstrating CD68+ histiocytes and CD34+ fibroblasts (Fig 2). Based on clinicopathologic correlation, a diagnosis of granulomatous scleromyxedema was made. Subsequently, the patient was started on dapsone (up to 100 mg daily), and topical ruxolitinib (1.5% cream). Patient applied topical ruxolitinib sparingly to his most bothersome sites and noted immediate improvement in pruritus across his upper portion of the back and ears. Ongoing use led to reduction in skin redness and induration, notably over the central chest, upper portion of the back, and ears (Fig 3).

**Fig 4:**
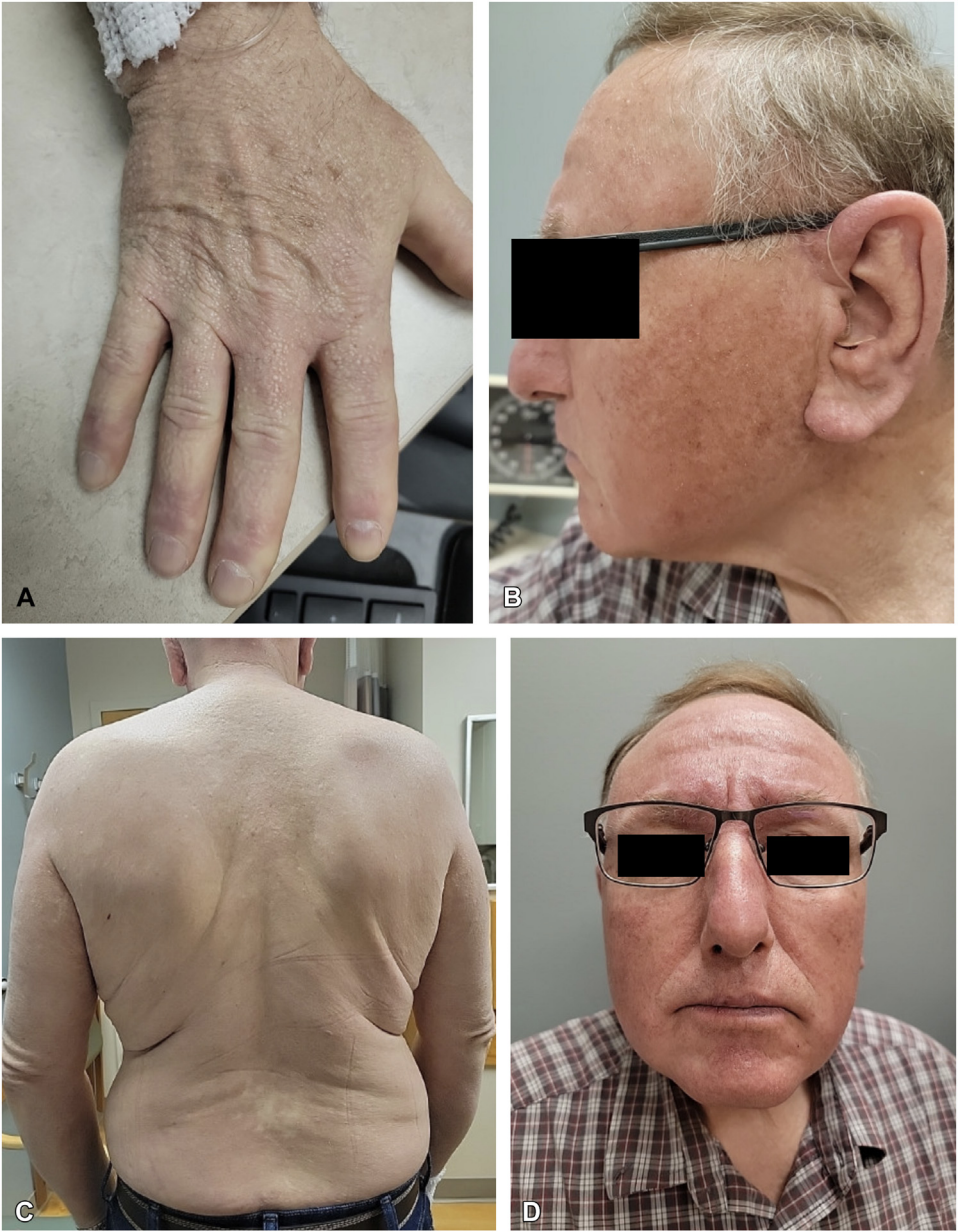
Further clinical improvement following treatment with intravenous immunoglobulin. **A**, Reduction in size and thickness of papules on the dorsal aspect of the hand. **B**, Reduction in number and size of mucinous papules, skin induration and textural changes, red coloration, and tenderness of his ears and face. **C**, Flattening of papules and plaques and resolution of erythema across his entire body, including his back. **D**, Reduction in induration of the forehead, glabella, and perioral face.

The patient was subsequently referred to hematology and oncology, at which time he underwent bone marrow biopsy. Bone marrow biopsy revealed a small clonal population (<5%) of lambda-restricted plasma cells with *t*(11,14) identified by fluorescence *in situ* hybridization, with no evidence of malignancy. The patient was initiated on IVIG 2 g/kg administered in divided doses every 28 days, with significant improvement of skin tightness, erythema, and texture across his entire body with improved mobility and resolution of pruritus 1 month after initial IVIG dose (Fig 4).

## Discussion

The diagnosis of SM requires correlation among clinical, histologic, and laboratory data. While the triad of mucin deposition, fibroblast proliferation, and fibrosis is classically seen on histology, a granulomatous variant of SM has been reported.^2^^,^^4^^,^^5^ In a retrospective study of 34 patients diagnosed with SM, 17.6% (6/34) of patient were found to have interstitial GA-like features on histology: diffuse interstitial proliferation of epithelioid cells with lymphocytes and giant cells arranged around vessels and between collagen fibers.^2^ For our patient, an initial histologic examination similarly revealed variably interstitial and vaguely palisaded histiocytic infiltrate consistent with a diagnosis of interstitial GA. However, clinical features (small, waxy papules with extensive facial involvement, particularly of the glabella and ears^6^) were discordant with typical GA and prompted additional laboratory investigation. The associated monoclonal gammopathy and other histopathologic features (mucin deposition, increased fibroblasts and CD68+ histiocytes) confirmed the diagnosis of granulomatous SM. Despite the potential for shared histopathologic features between GA and granulomatous SM, the 2 entities diverge in prognosis and approach to therapy. Suspicion for SM should prompt workup for underlying plasma cell dyscrasias and follow-up with hematology and oncology for the treatment of monoclonal gammopathy of clinical significance. As demonstrated in our case, cutaneous manifestations of SM are likely to be resistant to standard therapies used in GA such as topical steroids and narrow-band UV-B treatment.

In addition to the treatment effect from IVIG, which has been previously described,^6^ our patient noted significant symptomatic improvement 1 month following use of topical ruxolitinib and increased oral dapsone. Other reported treatments of scleromyxedema include systemic steroids and other immunomodulatory agents.^3^ Ruxolitinib is a JAKi used systemically to treat myeloproliferative neoplasms driven by overactivation of the JAK-signal transducers and activators of transcription pathway,^7^ and topically to treat atopic dermatitis.^8^ It is hypothesized that clinical manifestations of SM are due to an immunologic response to the underlying monoclonal gammopathy resulting in fibroblast stimulation and production of glycosaminoglycans.^2^ Although the causative factor remains unknown, *in vitro* studies have demonstrated that serum of SM patients may stimulate fibroblast proliferation.^9^ Animals studies with ruxolitinib have demonstrated antifibrotic effects within lungs of mice with bleomycin-induced pulmonary fibrosis, highlighting the use of JAKi as a potential therapeutic option for fibrotic disorders.^10^ Our case supports the use of topical JAKi therapy in addition to dapsone and IVIG for the treatment of cutaneous SM. We hypothesize that the clinical efficacy of topical JAKi in our patient relates to its antiinflammatory and antifibrotic activity in the skin, suggesting the possible role of JAK inhibition in other fibrotic skin conditions.

## Conflicts of interest

None disclosed.
